# Editorial: Cognitive hearing science: Investigating the relationship between selective attention and brain activity

**DOI:** 10.3389/fnins.2022.1098340

**Published:** 2022-12-13

**Authors:** Jerker Rönnberg, Anu Sharma, Carine Signoret, Tom A. Campbell, Patrik Sörqvist

**Affiliations:** ^1^Department of Behavioral Sciences and Learning, Linnaeus Centre HEAD, Linköping University, Linköping, Sweden; ^2^Department of Speech, Language and Hearing Sciences University of Colorado at Boulder, Boulder, CO, United States; ^3^Faculty of Information Technology and Communication Sciences, Tampere University, Tampere, Finland; ^4^Department of Building Engineering, Energy Systems and Sustainability Science, University of Gävle, Gävle, Sweden

**Keywords:** selective attention, divided attention, cognitive hearing science, brain activity, multi-modality, working memory, aging, brain imaging methods

## Introduction

“Everyone knows what attention is. It is the taking possession by the mind in clear and vivid form, of one out of what seem several simultaneously possible objects or trains of thought. Focalization, concentration, of consciousness are of its essence. It implies withdrawal from some things in order to deal effectively with others, and is a condition which has a real opposite in the confused, dazed, scatterbrained state which in French is called distraction, and Zerstreutheit in German,” (James, 1890, p. 403–404). How does such a psychological concept relate to human brain activity? An influential model in clinical neuropsychology (Sohlberg and Mateer, 2001) differentiates five dissociable components that are focused, sustained, alternating, selective, and divided attention. Sustained attention concerns focusing attention on stimulation for an extended period. Selective attention concerns concentrating on one source of information in exclusion of another, in the service of some task. Divided attention concerns attending to one task when there are other attentional demands, such as another parallel task. From a cognitive hearing science perspective, attention has been a somewhat nebulous concept that depends partially on working memory (Barrouillet and Camos, 2020; Rönnberg et al., 2022a,b) and upon related executive control mechanisms (Badre, 2021).

In the current topic, we include multi-modal attention studies employing a plethora of measures from several brain-imaging and behavioral techniques. The topic reveals how the field is developing, maturing, and diversifying. This collection assembles world-leading researchers' more exciting developments from groundbreaking experiments spanning the last seven years. This research not only provides us with new knowledge about attentional processes but also about the intricacies of perceptual-cognitive interactions.

Sörqvist et al. (2012) make a case for cognitive hearing and the early attentional-steering of auditory input: Visual working memory load dampens Wave V of the auditory brainstem response. Such a visual working memory load also targets auditory cortex (Sörqvist et al., 2016). Accordingly, conscious and intentional processing of stimuli, presented cross-modally, can penetrate modular brain functions performing auditory processing within the first few milliseconds of the onset of a sound (see also Ikeda and Campbell, 2021). Generally speaking, sensory and cognitive processing blend to a much larger extent than previously acknowledged (Rönnberg et al., 2022a,b). Cognitive hearing science's new early filter model explains the top-down influences upon early sensory processing in relation to existing corticopetal-corticofugal loops (Marsh and Campbell, 2016; Campbell and Marsh, 2018, 2019).

Selectively attending to perceived dialogue or sounds is vital for smooth communication processes. Factors that affect selective attention not only include the source of speech or nonspeech sound, hearing status, and the listener's motivation for attending to the sound, but also effort and listener fatigue (see Pichora-Fuller et al., 2016). Further, recent research investigating the attentional processing of speech is revealing other key factors that affect our selective attention. These new factors include: the quality of attended speech, semantic predictability, grammatical complexity, and the number of competing sources of speech, as well as whether the masker speech is in the listener's mother tongue or not. This Research Topic, therefore, gathers together studies investigating the effects of what Sohlberg and Mateer (2001) term sustained, selective, and divided attention upon brain regions that relate to the aforementioned cognitive and perceptual factors.

To foreshadow the ensuing editorial, on a simple level of analysis, supramodal cortical regions during audiovisual divided attention are not the neural equivalent of capacity-limited bottlenecks. However, selective attention to visual phonological material exhibits an intermodal character that affects the brain's representation of the auditory stimulus. Turning from a simple to a more complex communicative level, hemodynamic investigations characterize the different kinds of activation when selecting or dividing attention concerning auditory and visual modalities. Intriguing is how that division, particularly under adverse conditions, can compromise the activation of the social brain network. On this complex level, we bring you new EEG approaches to indexing listening effort and fluctuations in sustained attention, as has a future in brain-computer interfaces to dynamically steer the signal processing in hearing-assistive devices according to transient neurocognitive state. We then introduce you to how cognitive training can relatively rapidly re-calibrate the perceptual systems dealing with speech. The editorial then evaluates the successes of investigations that psychologically characterize inter-individual differences in attentional effects on hemodynamic measures of brain activity in special populations. These populations are not only of elderly individuals but also of adults with attention deficit hyperactivity disorder (ADHD). We conclude with how explicit intention can limit the cortical processing of predictable pitch change, arguably *via* brain processes relating to the top-down influence of auditory selective attention. The consensus in the field hitherto considered such cortical processing of pitch deviance as largely task-independent, if not preattentive and impenetrable to volition.

## Levels of analysis

### Simple levels

In a relatively early functional magnetic resonance imaging (fMRI) dual-task study centering upon divided attention (Salo et al.), participants concurrently attended to a time-varying series of spoken and written letters. Participants perform one of nine bimodal discrimination tasks, which had a visual and an auditory task component. Either the auditory or visual task component, or both, could concern phonological features, discriminating between whether letters had a *name* starting with a vowel or a *name* starting with a consonant. Either task component, or both, could concern spatial features, discriminating whether the stimulus was on the left or right. Either task component, or both, could also concern simple features, judging the gender of the voice or the font of the letter. Of the nine tasks, the baseline dual task, with which to compare the other dual tasks, involved discriminating the simple auditory feature of gender of voice whilst discriminating the simple visual feature of font. This baseline dual task had no spatial or phonological requirements. A prior study provided the corresponding unimodal single discrimination task data with which to also separately compare the other dual tasks.

Comparison of dual tasks with the baseline dual task revealed different supramodal patterns of activation in the left medial frontal gyrus and right inferior parietal lobule. These findings juxtapose with how these supramodal activations were absent in the comparison with the single task components. The interpretation offered was that supramodal phonological and spatial areas are similarly activated during single tasks requiring phonological or spatial processing in one modality as during dual tasks that require: (i) both auditory and visual phonological processing implicating the left medial frontal gyrus or (ii) both auditory and visual spatial processing implicating the right inferior parietal lobule. These supramodal regions are thus arguably not the seat of some phonological or spatial capacity limitation serving as a bottleneck at the confluence of auditory and visual information.

In an electroencephalographic (EEG) investigation, Alho et al. reveal that the frequency-following response, which to-be-ignored heard distractor syllables elicit, goes relatively unaffected by a primary cross-modal task: Across two different heard syllables, whether that task is either a more challenging phonological task or a non-phonological task, on which performance is faster and more accurate, this response's amplitude does not differ significantly. This frequency-following response phase-locks to the vowel's acoustical fundamental.

As an editorial aside, at first, this null effect of the to-be-attended task seems uncontentious for the notion that any biased competition (Desimone and Duncan, 1995) between distractor and target during selective attention confines to intramodal filtering (Parks et al., 2011). Such a notion thus assumes the independence of modality-specific visual and auditory processing resources. Accordingly, there are no cross-modal effects on frequency-following responses (Szychowska and Wiens, 2021). However, deeper scrutiny of Alho et al.'s data is not so uncontentious for this notion.

The deviance of a rare unexpected syllable, interspersed amidst a sequence of repeated standard syllables in an oddball sequence, can cause the frequency-following response's amplitude to be higher. The presence of this effect of deviance seems to depend upon the acoustical content of the standard-deviant pairing in the oddball sequence of distractors. Crucially, when that pairing is sufficient for an effect of deviance, the extent of the effect proves higher when the primary task is phonological rather than nonphonological. Such a task-dependent influence may well result from the more demanding cross-modal phonological task either augmenting the frequency-following response to the deviant, or suppressing the corresponding response to the standard, or both. In either case, as a further editorial aside, this task-dependent influence is difficult to reconcile theoretically with an independence of visual and auditory processing resources during selective attention such that there are no cross-modal effects on frequency-following responses (Szychowska and Wiens, 2021).

Alho et al. do postulate a top-down modulation of activity in subcortical structures *via* corticofugal connections descending from the auditory cortex, as does cognitive hearing science's new early filter model (Marsh and Campbell, 2016; Campbell and Marsh, 2018, 2019). Alho et al.'s task-dependent influences upon frequency-following response phenomena are in more accord with this model than an independence of visual and auditory processing resources during selective attention. Although Alho et al. do demonstrate that the deviant syllable elicits a mismatch negativity, there is no analogous significant task-dependence of the amplitude of this component that could have functionally unrelated cortical generators. While it could thus be tempting to consider the task-dependent influence on frequency-following responses as purely subcortical, Alho et al.'s stimuli arguably also engage cortical generators (Coffey et al., 2019). These generators are distinct from that of the auditory mismatch negativity and are capable of tracking modulation frequencies upto 200 Hz (Brugge et al., 2009; Nourski et al., 2013).

In a cross-modal study of a slightly different sort, Nuernberger et al. investigate how different forms of noise influence the processing of tactile stimuli during a mechanical detection threshold task. The results show that whereas unpleasant everyday noise, “real noise”, leads to an increased tactile sensitivity, white noise impairs such tactile sensitivity. Significant differences in brain activity and connectivity in distributed networks accompany this interaction between acoustic and tactile stimuli. Rather than invoking notions of selective and divided attention, the interpretation that the authors offer is that real noise creates a brain state for enhanced unimodal processing of tactile stimuli as could be favored by “phasic attention” (Schlittmeier et al., 2015). In juxtaposition, white noise increases both activity and connectivity in the auditory and somatosensory cortices, the association cortex, and the thalamus. Such white noise thereby impairs tactile sensitivity cross-modally as could relate to selective attention.

### Complex levels

Moisala et al., in a semantic sentence congruency task, compared activations: (i) selectively attending to only the visual modality with (ii) selectively attending to only the auditory modality with (iii) divided attention on a bimodal version of that task. This task activates left prefrontal cortex activity in selective attention conditions, whereas the same areas showed significant activity increases during divided attention. The results suggest that divided attention tasks interfere with each other, stimulating increased activity in the same cortical areas without any compensatory activity in other cortical areas. The cost is therefore lower performance in the divided attention tasks.

The fMRI studies of Salo et al. and Moisala et al. both show that comparing selective with divided attention tasks reveal different brain activation patterns (see also Salo et al., 2017). However, neither study clarifies whether the division of attentional tasks rely upon cross-modal or within-modality interference. Leminen et al., in an ecologically compelling investigation, demonstrate that selective attention to dialogues activates not only areas in brain networks for audiovisual speech processing and understanding, but also a social brain network. What social knowledge that we use and gain in a conversational situation depends not only upon what we can see, hear, and already know, but also upon how we integrate the information from the two modalities, to an extent pre-attentively, when one modality is less informative. As well, this knowledge depends upon our mental flexibility to direct intermodal selective attentional resources to alternate between the auditory and visual modality. Arguably, with hearing-assistive devices that have a limited number of channels, kindred to Leminen et al.'s signal processing, the temporal fine structure of the audio is a valuable source of information in that process that determines audiovisual quality. Reductions in audiovisual quality increase the demands upon selective attention that could relate to Leminen et al.'s observed fronto-parietal activation. Such pathfinding studies pave the future for a cognitive hearing science that shall determine how intermodal brain processes glean socially relevant meanings from heard utterances in audiovisual contexts under adverse conditions.

Along the lines of more complex communicative levels of analysis, Jaeger et al.'s participants attended to one story in the (competing) presence of another. Analysis of intra-individual variation in EEG-decoding performance over time relates to behavioral performance together with subjective ratings of listening effort, motivation, and fatigue. Parameters describing the individual performance indicated significant differences in EEG-decoding performance over time, which closely related to the behavioral performance in the selective listening task. Those fluctuations could have implications for the control of hearing-assistive devices *via* a brain computer interface in multi-talker situations.

Shahsavari Baboukani et al. identified an EEG-based measure of alphaband phase synchrony that arguably indexes listening effort. This investigation measures the EEG of aided listeners with hearing impairments during a continuous speech-in-noise task under conditions of background noise and a competing talker. This study shows that the activation of noise-reduction schemes in hearing aids can non-linearly reduce listening effort in the parietal region-of-interest. Indeed, the authors propose that the investigation of the phase synchrony within regions-of-interest over the scalp can reflect the effects of hearing aids in hearing-impaired individuals under ecological listening conditions.

## Training

A 15-min period of audiovisual spatial training (Hanenberg et al.) affects participants' audio-spatial performance on a selective attention task. The task used by Hanenberg et al. requires selecting the auditory target from different positions of three distractor words. Training affected the amplitude of the N2 deflection of the event-related potential (ERP), which is known to index auditory spatial attention. The N2 is significantly higher in amplitude after audiovisual-congruency training compared with other feedback or incongruous training conditions. This finding was apparent for younger, yet not older, participants. These findings arguably offer insights into the cross-modal processes that audiovisual-congruency training alters under “cocktail-party” conditions. This short-term alteration results in enhanced correlates of auditory selective spatial attention. Focusing on the very limited time necessary to improve neural and behavioral performance, the results by Hanenberg et al. are in accord with the independent study of Moradi et al. (2019)—Brief exposure to audiovisual stimulus materials improves performance on auditory perception tasks. This finding generalizes to tasks ranging from the simple auditory gating of vowels and consonants to sentence perception in noise. Moradi et al. (2019) dub this phenomenon “perceptual doping”. The brain seems to have the power to rapidly re-calibrate the perceptual systems dealing with speech.

## Attention in special populations

### Attention ADHD

ADHD per definitionem affects sustained attention and the attention networks of the brain. Although distractibility is not definitively at the core of this syndrome, selective attention can be an issue (Pelletier et al., 2016). Two investigations by Blomberg et al. demonstrate how cognitive and attention networks interact. One of the principal findings of Blomberg, Johansson Capusan, et al. is that under cognitive load, in a visual working memory task, the attention networks tend to become “blended” to a larger extent when working memory is put under stress. Results indicate that adults with ADHD, compared to controls, cannot attenuate auditory cortical responses to the task-irrelevant sound when working memory demands is high (i.e., as in a 2-back version of an n-back task). Further, heightened auditory activity to task-irrelevant sound correlates significantly with both poorer working memory performance and symptomatic inattentiveness. As already shown by Blomberg et al. (2019), a behavioral composite latent working memory capacity measure could predict performance in different kinds of degraded/noise conditions. Finally, in a resting state study (Blomberg, Signoret, et al.), the default mode network still interacts more with the ventral and auditory attention networks for adults with ADHD relative to controls, as arguably compromises selective attention causing higher levels of distraction from auditory stimuli.

### Attention and aging

Schneider et al.'s EEG study revealed that, in young adults, there is an increase in the early cortical activity generating the N1 and P2 ERP deflections to a word in babble with longer masker onset delays. This cortical activity in older adults goes unaffected by that delay. These results support the hypothesis that an increase in onset delay improves stream segregation in younger adults in both noise and babble. The results also support the hypothesis that this improvement occurs only in noise for older adults. These influences upon stream segregation are also evident in early cortical processes.

As an editorial comment, one may wonder what a cognitive interpretation of these effects may look like. The ability to segregate competing speech signals is dependent on working memory, while the ability to process a speech signal that competing background (non-speech) noise masks is less dependent upon working memory (Sörqvist and Rönnberg, 2012). Pertinent are the age-related differences in working memory capacity (Wingfield et al., 1988) and the role of that capacity in temporal discrimination and temporal processing (Broadway and Engle, 2011). As such, differences in working memory capacity could thus explain the age-related differences in the ability to make use of the onset delay to support auditory stream segregation in speech noise. One possibility is that high working memory capacity is a prerequisite for detecting, accessing, and utilizing the temporally fine-tuned information necessary for the segregation of the two speech streams.

## Intentional-explicit influence on attentional processing

Widmann and Schröger employed an oddball paradigm to investigate intention-based predictive or non-predictive processing of standard and pitch-deviant sounds. The manipulation of predictive processing was that the participant either heard a completely unpredictable oddball sequence or, on-the-fly, had partial control of the oddball sequence: The participant's task was pressing one button occasionally to produce a predicted deviant tone in the sequence, whereas pressing another frequently produces a predicted standard tone, albeit occasionally the system for stimulus presentation randomly produced a mispredicted deviant instead of that predicted standard. Intriguingly, both unpredictable deviants and mispredicted deviants elicited the auditory mismatch negativity ERP component, but predicted deviants did not. This elegant procedure shows that intention-based prediction, which relies upon the top-down influence of the action intention prediction, attenuates this mismatch negativity. Thus, even though the predicted deviant violates an auditory regularity, brain processes that relate to top-down cognitive predictions limit the generation of the mismatch negativity. While the sensory-memory trace hypothesis has fallen from grace and these new findings are difficult to reconcile with the adaptation hypothesis, a role for working memory capacity is not an assumption of the predictive-coding account proliferating extant explanations of the auditory mismatch negativity findings. Widmann and Schröger's findings dovetail with corroborative evidence of a somewhat different sort concerning the investigation of predictability: Semantic cues associated with target sentences prime performance on a speech-perception-in-noise test in which those sentences are stimuli (Zekveld et al., 2013). Whereas working memory capacity predicted the extent of this priming effect, an intriguing open question is how that capacity relates to Widmann and Schröger's influence of prediction.

## Closing

In sum, this topic's collection of papers explores the relationship between factors affecting different forms of attention and brain activity, as well as the brain regions that competing audio and audio-visual cues or sources activate. To evaluate, these papers, together, succeed in substantially advancing cognitive hearing science's understanding of human attention and the related brain processes. Having attracted mostly multi-modal investigations, our overwhelming impression from re-reading the articles is that this topic now sets the scene for new avenues in cognitive hearing science: This new avenue shall usefully determine how attention relates to the intermodal brain processes that operate when people extract meaning under adverse conditions.

## Author contributions

JR drafted the editorial. AS, CS, TC, and PS contributed to the final submitted version. All authors contributed to the article and approved the submitted version.
